# Tea Disease Detection Method Based on Improved YOLOv8 in Complex Background

**DOI:** 10.3390/s25134129

**Published:** 2025-07-02

**Authors:** Junchen Ai, Yadong Li, Shengxiang Gao, Rongsheng Hu, Wengang Che

**Affiliations:** 1 Faculty of Information Engineering and Automation, Kunming University of Science and Technology, Kunming 650500, China; ajc5454@163.com (J.A.);; 2 School of Data Science and Engineering, Kunming City College, Kunming 650101, China; 3 School of Artificial Intelligence and Computer Science, Jiangnan University, Wuxi 214122, China

**Keywords:** deep learning, detection of tea diseases, convolutional neural network, target detection, leaf disease

## Abstract

Tea disease detection is of great significance to the tea industry. In order to solve the problems such as mutual occlusion of leaves, light disturbance, and small lesion area under complex background, YOLO-SSM, a tea disease detection model, was proposed in this paper. The model introduces the SSPDConv convolution module in the backbone of YOLOv8 to enhance the global information perception of the model under complex backgrounds; a new ESPPFCSPC module is proposed to replace the original spatial pyramid pool SPPF module, which optimizes the multi-scale feature expression; and the MPDIoU loss function is introduced to optimize the problem that the original CIoU is insensitive to the change of target size, and the positioning ability of small targets is improved. Finally, the map values of 89.7% and 68.5% were obtained on a self-made tea data set and a public tea disease data set, which were improved by 3.9% and 4.3%, respectively, compared with the original benchmark model, and the reasoning speed of the model was 164.3 fps. Experimental results show that the proposed YOLO-SSM algorithm has obvious advantages in accuracy and model complexity and can provide reliable theoretical support for efficient and accurate detection and identification of tea leaf diseases in natural scenes.

## 1. Introduction

China, the birthplace of tea, boasts a rich tea culture deeply cherished by its populace [1]. The nation attaches great importance to the preservation and advancement of tea culture, actively promoting the high-quality development of the tea industry [2]. In this context, ensuring high quality and yield in tea production is particularly crucial [3]. However, tea diseases significantly impact both yield and quality [4]. Therefore, research into tea disease detection holds substantial practical significance for the tea industry. Efficient and timely identification of these diseases is critical for prompt intervention to prevent widespread crop damage, making real-time or near real-time detection capabilities highly desirable for on-site diagnosis using mobile devices or for rapid.

Traditional tea disease detection relies on the visual expertise of tea farmers, a method prone to misjudgment and omission, suffering from low efficiency and high subjectivity. With the evolution of computer vision [5], plant disease detection technologies have been widely adopted in agriculture [6], including machine learning methods such as SVM (Support Vector Machines) [7] and K-NN (K-Nearest Neighbor) algorithms [8]. For instance, Sun et al. [9] proposed an SVM-based tea disease detection method. While these machine learning algorithms overcome the misjudgment potential inherent in manual diagnosis and reduce resource demands, their efficacy largely depends on manually designed features and requires careful feature selection. Consequently, achieving high recognition accuracy is challenging in noisy scenarios, such as those with uneven illumination.

The advent of deep learning has propelled significant progress in plant disease detection technology [10]. Deep learning-based plant disease detection models are broadly categorized into three types. The first encompasses basic image classification models, such as AlexNet [11], ResNet [12], DenseNet [13], and other CNN (Convolutional Neural Network) architectures. For example, Muh Hanafi et al. [14] proposed an automatic tea disease detection method based on the ResNet architecture. These models offer significant advantages in feature extraction, transfer learning, and performance improvement, but also present shortcomings regarding computational resources, overfitting risks, and model complexity.

The second type includes two-stage object detection models, such as R-CNN [15], Faster R-CNN [16], and their derivatives. For example, in 2022, Chen et al. [17] developed a tea disease and insect pest recognition framework using Faster R-CNN, and Li et al. [18] proposed a tea disease detection model combining Mask R-CNN with wavelet transform. While these two-stage algorithms can detect leaf diseases more accurately, their large network models and slow detection speeds result in poor practical application performance.

The third category comprises single-stage object detection models, like SSD (Single Shot Multibox Detector) [19], YOLO (You Only Look Once) [20], and their improved versions. For instance, Li et al. [21] introduced a lightweight tea disease recognition system based on MobileNetV3, and Xue et al. [22] proposed the YOLO-Tea model, an enhancement of the YOLOv5 architecture. Both studies indicate that existing methods still have room for improvement in feature expression and cross-scenario adaptability. In 2024, Ye et al. [23] proposed YOLOv8-RMDA, a lightweight, high-precision model based on an improved YOLOv8 architecture. By incorporating the MixSPPF hybrid pooling module, RFCBAM attention mechanism, a dynamic detection head, and an IoU loss function, they effectively addressed the trade-off between accuracy and real-time performance for tea leaf disease detection in complex backgrounds while also reducing model complexity. The YOLO series, in particular, has gained prominence for its effective balance between speed and accuracy, with lightweight variants like YOLOv8n providing a promising baseline for developing efficient models suitable for deployment on resource-constrained edge devices, a key consideration for practical agricultural applications.

Single-stage detectors like YOLO provide an effective balance of speed and accuracy, yet the computer vision landscape is rapidly advancing with architectures like Vision Transformers (ViTs) and Foundation Models excelling on general benchmarks. However, their application in specialized domains such as agricultural disease detection is often hindered by substantial computational and data requirements, particularly for edge deployment. Thus, optimizing efficient, adaptable architectures like the YOLO series for domain-specific challenges remains a crucial research direction for field-deployable solutions; this study focuses on enhancing YOLOv8 for this purpose.

Current tea disease research often overlooks detection in complex natural environments, where model generalization is severely impacted by issues unique to tea cultivation: Frequent leaf occlusion within dense canopies, feature ambiguity from uneven illumination on glossy leaves, and missed detection of minute, early-stage lesions critical for timely management. These acute challenges necessitate tailored optimizations beyond general-purpose models.

This study employs object detection to identify and localize tea diseases. While real-time semantic segmentation has become increasingly viable with state-of-the-art models [24], our choice of object detection was guided by the specific goals and practical constraints of this project. The primary objective of rapid disease identification and localization is effectively met by the bounding box outputs. Critically, the significantly lower annotation workload for creating bounding boxes, compared to the pixel-wise masks required for segmentation, made the development of our large-scale, multi-class ‘Tea Datasets’ feasible. Therefore, object detection offered the most practical and scalable pathway to develop a robust model for this application, balancing actionable output with development cost and maintaining efficiency for potential on-device use.

Therefore, to achieve rapid and accurate identification of tea diseases in complex backgrounds, this paper proposes a novel improved YOLOv8 model, YOLO-SSM (YOLOv8 incorporating SSPDConv, ESPPFCSPC, and MPDIoU). The novelty of this work lies not in the creation of entirely new fundamental algorithms, but in the strategic and synergistic integration of specifically adapted components designed to optimize performance for this particular domain. This includes the introduction of an SSPDConv (SPD-SimAM-Convolution) module, which is integrated into the YOLOv8 backbone to enhance global information extraction, thereby improving the model’s recognition capability in complex backgrounds. Concurrently, the ESPPFCSPC (Enhanced Spatial Pyramid Pooling Fast with Cross Stage Partial Connection) module is introduced to replace the original Spatial Pyramid Pooling module, extracting data features through dual channels to enhance feature representation quality and address problems of feature loss under occlusion and feature ambiguity from uneven illumination. Furthermore, the MPDIoU (Multi-Point Distance Intersection over Union) loss function is employed to improve the localization and recognition of small lesion areas, tackling the issue of missed small target detections. Finally, the improved YOLOv8n model is trained on self-made and public datasets to validate its detection performance.

## 2. Model Architecture and Improvements

### 2.1. YOLOv8 Architecture

**Figure 1:**
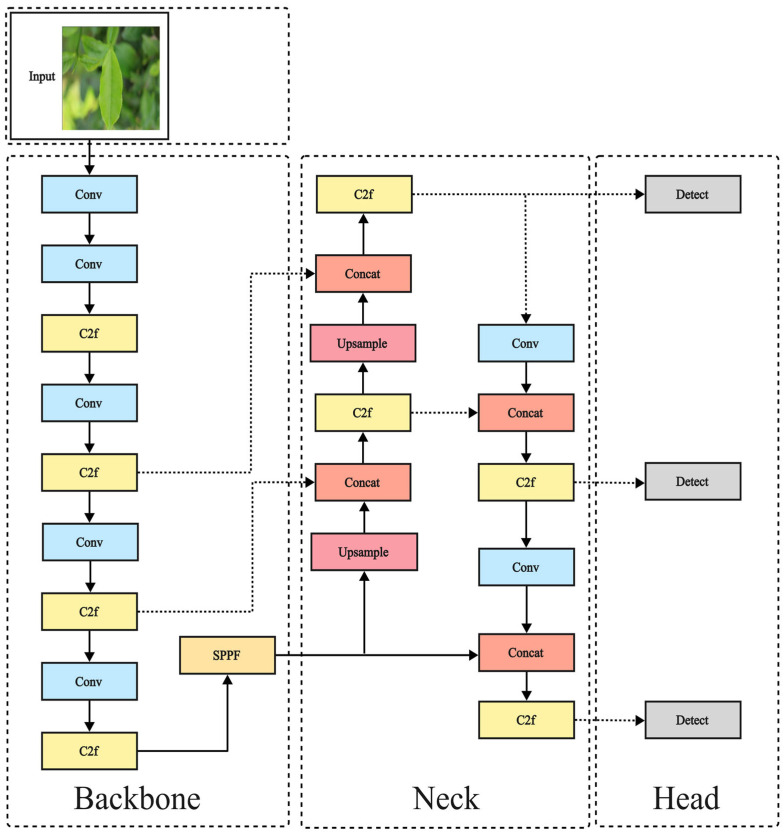
YOLOv8 network structure.

The YOLO series, a benchmark in single-stage object detection, innovatively reframes target localization and classification as regression problems. This approach enables a unified network architecture to simultaneously output bounding box coordinates and class probabilities, overcoming limitations of traditional methods by maintaining high accuracy alongside rapid processing speeds. YOLOv8 [25] represents a recent and stable iteration within this series. As illustrated in Figure 1, its architecture typically comprises a backbone for feature extraction (incorporating modules such as Conv (Convolution) [26], C2f (Cross Stage Partial 2 with Focus), and SPPF (Spatial Pyramid Pooling Fast) [27]), a neck (utilizing FPN (Feature Pyramid Network) [28] and PANet (Path Aggregation Network) [29]), and detection heads. Key advancements in YOLOv8 over its predecessors include the integration of C2f and SPPF modules within the backbone. These modules enhance feature extraction capabilities and the ability to capture salient features, thereby improving overall model performance and robustness. The lightweight YOLOv8n variant offers a strong feature extraction foundation with a favorable speed-performance trade-off, making it suitable for real-time agricultural applications on edge devices and an excellent platform for targeted improvements. Therefore, YOLOv8n is adopted as the baseline model for this research.

### 2.2. YOLO-SSM Tea Disease Detection Model

**Figure 2:**
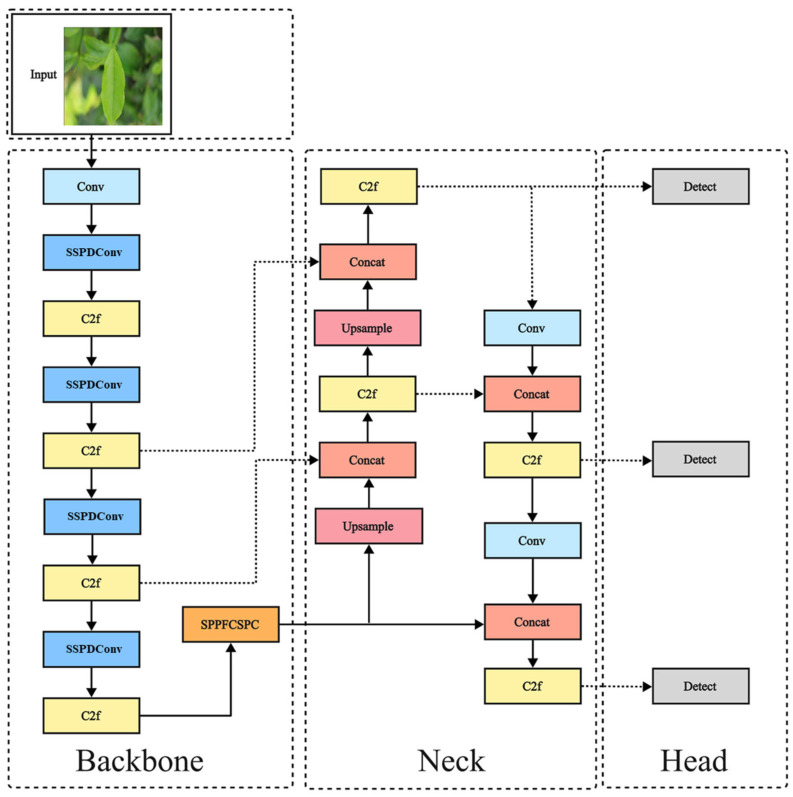
YOLO-SSM network structure.

Building upon the YOLOv8 architecture, this study proposes YOLO-SSM, an improved model specifically designed for tea disease detection. To enhance detection performance under the challenging conditions encountered in tea plantations, YOLO-SSM incorporates several key modifications: The SSPDConv convolution module is introduced to bolster feature extraction, particularly for discerning subtle disease patterns amidst complex leaf textures and under variable illumination common in field imagery; an improved spatial pyramid pooling module, ESPPFCSPC, is adopted to optimize feature fusion, crucially for recovering features lost due to dense tea leaf occlusion and mitigating ambiguity from uneven illumination characteristic of tea leaves; and the MPDIoU loss function is employed to refine localization accuracy, especially for the critical task of identifying early-stage, small disease lesions vital for effective crop management. These strategic enhancements collectively aim to achieve high detection performance while effectively managing the increase in model parameters. The overall architecture of YOLO-SSM is depicted in Figure 2.

#### 2.2.1. Optimizing the Convolution Module

The standard Conv module employed in YOLOv8 efficiently extracts local spatial features and offers considerable computational power. However, its inherent focus on local information can lead to overlooking distant target details. Furthermore, conventional pooling operations within such modules often result in the loss of fine-grained edge details, thereby impairing detection accuracy. Moreover, its rigid structure struggles to flexibly capture spatial feature variations across an image, limiting overall model adaptability.

To address these limitations, this paper introduces SSPDConv, a novel convolutional module. The core of SSPDConv builds upon the principles of SPDConv [30], which utilizes an SPD (space-to-depth) transformation, replacing traditional stride convolution and pooling layers. This approach effectively preserves fine-grained image details and mitigates information loss, which is particularly crucial for tea disease detection. It enables more accurate identification of critical features, such as subtle, early-stage lesions that are often difficult to distinguish from complex leaf textures, especially under challenging conditions like insufficient illumination, blur, or low resolution.

**Figure 3:**
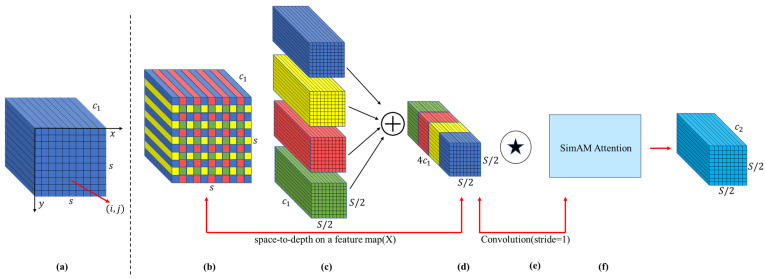
The calculation process within the SSPDConv module. The steps are: (**a**) receiving an input feature map; (**b**–**d**) applying a Space-to-Depth (SPD) transformation to preserve spatial details while increasing channel depth; (**e**) using a 1×1 convolution to adjust channel dimensions; and (**f**) applying the SimAM attention mechanism to recalibrate features and produce the final output. In this diagram, the + symbol denotes feature concatenation along the channel dimension, and the ★ symbol denotes a convolution operation.

**Figure 4:**
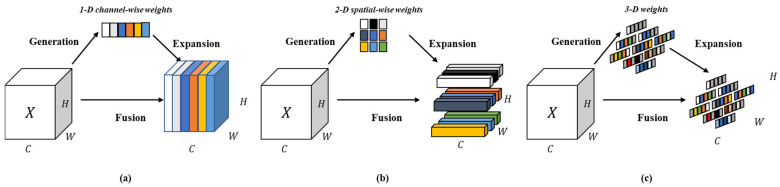
Principles of Attention Mechanisms: (**a**) Channel-wise Attention, (**b**) Spatial-wise Attention, and (**c**) Full 3D Attention (SimAM Principle) as utilized in SSPDConv.

Building upon SPDConv, this work strategically integrates the SimAM (A Simple, Parameter-Free Attention Module) [31] attention mechanism. SimAMs parameter-free dynamic weight allocation enhances the expressive power of disease-related features while concurrently suppressing interference from complex backgrounds. In the context of tea disease detection, this capability is vital for mitigating the obscuring effects of the tea plant’s intricate leaf textures, variations in illumination, and shadows, which can otherwise mask subtle lesion characteristics. Thus, the synergistic design of SSPDConv, combining the detail preservation of SPD with the feature refinement of SimAM, empowers the model to more effectively address the multifaceted challenges prevalent in tea disease detection, such as identifying minute early-stage lesions, distinguishing features amidst complex leaf textures and variable illumination, and enhancing robustness against background noise. The SSPDConv convolutional process is illustrated in Figure 3, while the principle of the SimAM attention mechanism is depicted in Figure 4.

The operational flow of the SSPDConv module, as illustrated in Figure 3, comprises the following sequential steps:

- Input Feature Map (Figure 3a): The module receives an input feature map of dimensions S×S×C1, where S denotes the spatial resolution (height and width) and C1 represents the initial number of channels.
- Space-to-Depth Transformation (Figure 3b–d): The input feature map first undergoes an SPD transformation. This operation reorganizes spatial information into the channel dimension by performing strided sampling (effectively taking pixel blocks, as illustrated conceptually from Figure 3b,c to the merged representation in Figure 3d) and concatenating the resulting sub-maps. Consequently, the spatial resolution is reduced from S×S to (S/2)×(S/2), while the number of channels expands from C1 to 4C1.
- 1 × 1 Convolutional Operation (Figure 3e): The feature map resulting from the SPD transformation, now with dimensions (S/2)×(S/2)×4C1, is subsequently processed by a 1×1 convolutional layer. This layer primarily serves to adjust channel dimensionality and perform a linear recombination of features. In this implementation, the 1×1 convolution reduces the number of channels from 4C1 to C2, while the spatial dimensions (S/2)×(S/2) remain unchanged due to the 1×1 kernel and a stride of 1. The feature map size thus becomes (S/2)×(S/2)×C2.
- SimAM Attention Mechanism (Figure 3f): Finally, the feature map from the 1×1 convolution, with dimensions (S/2)×(S/2)×C2, is fed into the SimAM attention module. The SimAM module calculates a parameter-free energy function to evaluate the importance of each neuron, thereby generating 3D attention weights. These weights recalibrate the features, enhancing task-relevant key feature expressions while suppressing irrelevant information or noise from complex backgrounds. As SimAM typically preserves the input feature map’s dimensions, its output remains (S/2)×(S/2)×C2. This attention-recalibrated feature map constitutes the final output of the SSPDConv module.

Figure 4 illustrates the conceptual underpinnings of the attention mechanism employed within the SSPDConv module, highlighting its comprehensive approach to feature refinement. The figure contrasts simpler attention strategies with the more holistic 3D attention akin to that generated by SimAM.

- Channel-wise Attention: This panel conceptually depicts channel attention, where the mechanism learns to assign different importance weights to different channels of the input feature map X. This addresses ‘what’ features are more relevant by selectively amplifying or suppressing entire feature channels based on their global context. The weights (represented by the colored bar) are typically 1D and applied uniformly across the spatial dimensions of each channel.
- Spatial-wise Attention: This panel illustrates spatial attention, which focuses on ‘where’ the informative parts of the feature map are located. For each channel, a 2D spatial attention map (represented by the colored grid) is generated, highlighting salient regions and suppressing less important areas within that specific feature channel.
- Full 3-D Weights for Attention (SimAM Principle): This panel conceptually represents the approach adopted by mechanisms like SimAM, which is integrated into our SSPDConv. Instead of separately computing channel and spatial weights, SimAM directly infers 3D attention weights for each individual neuron within the input feature tensor X (H×W×C). It achieves this by defining a parameter-free energy function based on the linear separability of each neuron. Neurons exhibiting lower energy receive higher attention weights. This results in a fine-grained, 3D attention map that simultaneously considers channel-wise and spatial-wise importance at a neuron level. The output is a recalibrated feature map where each feature is adaptively refined based on its individual significance, leading to enhanced representation of critical information and suppression of noise or irrelevant background details.

#### 2.2.2. Optimizing the Spatial Pyramid Pooling Structure

The SPPF module, integrated into YOLOv8, effectively amalgamates multi-scale contextual information by serially stacking maximum pooling layers of varying sizes. This design enhances the model’s robustness in detecting targets across different scales. However, its reliance on multiple pooling operations can lead to the loss of local detail information, consequently diminishing detection accuracy. Furthermore, in the natural settings characteristic of tea cultivation, which frequently exhibit uneven illumination, severe inter-leaf occlusion, and complex backgrounds, SPPFs fixed pooling kernel sizes hinder its ability to dynamically adapt to target scale variations, and its capacity to extract non-uniformly distributed features is consequently limited.

To address these shortcomings, this paper proposes ESPPFCSPC, a novel pyramid pooling structure derived from SPPFCSPC [32]. SPPFCSPC builds upon SPPF by incorporating a CSP (Cross Stage Partial) architecture. This CSP structure mitigates computational redundancy by partitioning the feature map into two segments that are processed independently, thereby preserving rich feature information. Consequently, SPPFCSPC achieves superior integration of multi-scale feature information, enhancing the model’s ability to detect targets of varying sizes in complex backgrounds and improving inference speed while maintaining high accuracy. The CSP architecture also bolsters feature transfer and reuse by connecting features across stages. This effectively prevents information loss and significantly improves the model’s capacity to capture fine-grained target details, providing a distinct advantage in handling incomplete lesion features due to inter-leaf occlusion in dense tea canopies and mitigating feature ambiguity caused by uneven illumination on tea leaf surfaces.

**Figure 5:**
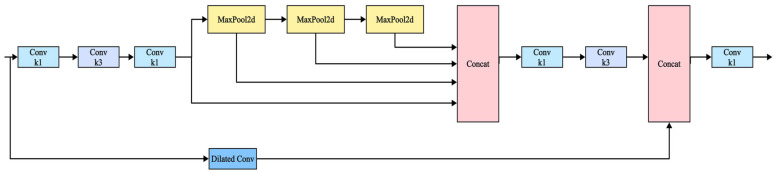
ESPPFCSPC network structure.

Building upon the SPPFCSPC framework, this paper introduces a key modification: The standard 1 × 1 convolution within the original SPPFCSPC branches is replaced with dilated convolution. This substitution aims to effectively expand the receptive field and capture broader spatial context information without substantially increasing computational load. This design choice is particularly beneficial for tea disease detection, as it enables ESPPFCSPC to more proficiently process the varied scales of disease presentation and subtle lesion characteristics often encountered amidst complex backgrounds with challenging light and occlusion conditions, thereby overcoming the receptive field limitations inherent in standard convolution. The network architecture of ESPPFCSPC is illustrated in Figure 5.

#### 2.2.3. Loss Function Design

The YOLOv8n model employs the CIoU (Complete Intersection over Union) loss function for bounding box regression. While CIoU considers multiple factors, including the distance between box centers and aspect ratios, its calculation is relatively complex, leading to increased computational overhead, particularly when processing a large number of targets. Furthermore, CIoUs sensitivity to aspect ratio, though intended as an improvement, can occasionally result in unstable loss values, especially in scenarios with significant variations in target shapes. During training, CIoU may also be prone to converging to local optima, particularly with inaccurately labeled or noisy data, potentially preventing the model from achieving optimal performance. Moreover, when dealing with small targets, CIoU might not effectively capture sufficient detail, thereby impacting detection efficacy. The CIoU calculation formula is as follows:

The CIoU loss function $L_{CIoU}$ (a scalar value) is defined as

(1) $$L_{CIoU}=1-IoU\left(A,B\right)+\frac{\rho^{2}\left(A_{ctr},B_{ctr}\right)}{c^{2}}+\alpha v$$

The penalty term $R_{CIoU}$ (a scalar value) within this loss is given by

(2) $$R_{CIoU}=\frac{\rho^{2}\left(A_{ctr},B_{ctr}\right)}{c^{2}}+\alpha v$$

where:

- A represents the predicted bounding box and B represents the ground-truth bounding box.
- $IoU\left(A,B\right)$ is the scalar value of Intersection over Union between boxes A and B.
- $A_{ctr}$ and $B_{ctr}$ denote the center points (vectors) of boxes A and B, respectively.
- $\rho^{2}\left(A_{ctr},B_{ctr}\right)$ is a scalar representing the squared Euclidean distance between the center points $A_{ctr}$ and $B_{ctr}$.
- c is a scalar representing the diagonal length of the smallest enclosing box that covers both A and B.
- α is a scalar positive trade-off parameter.
- v is a scalar value that measures the consistency of the aspect ratios.
- The terms v and α are defined as

(3) $$v=\frac{4}{\pi^{2}}{\left(\tan^{-1}\frac{w^{gt}}{h^{gt}}-\tan^{-1}\frac{w}{h}\right)}^{2}$$

(4) $$\alpha =\frac{v}{\left(1-IoU\right)+v}$$

In these formulas:

- (w, h) are scalar values representing the width and height of the predicted box A.
- ($w^{gt}$, $h^{gt}$) are scalar values representing the width and height of the ground-truth box B.

To address these limitations, this paper adopts the Multi-Point Distance Intersection over Union (MPDIoU) loss function [33]. MPDIoU enhances the evaluation of the relationship between predicted and ground-truth boxes by incorporating distance information from multiple points, thereby improving sensitivity to target position. By considering multi-point distances, MPDIoU generally exhibits greater robustness when handling complex scenarios such as leaf occlusion and can better accommodate target shape variations. Notably, MPDIoU demonstrates superior performance in small object detection by reducing reliance on a single bounding box through multi-point evaluation, enabling the model to capture finer details of small objects. Additionally, MPDIoU is more likely to converge to a superior solution during training, especially when the dataset contains noise. This is of paramount importance for accurately identifying minute, early-stage tea disease lesions, facilitating timely detection and intervention. Therefore, this research replaces the CIoU loss function with MPDIoU. Its calculation formula is as follows:

(5) $$d_{1}^{\;2}={\left(x_{1}^{prd}-x_{1}^{gt}\right)}^{2}+{\left(y_{1}^{prd}-y_{1}^{gt}\right)}^{2}$$

(6) $$d_{2}^{\;2}={\left(x_{2}^{prd}-x_{2}^{gt}\right)}^{2}+{\left(y_{2}^{prd}-y_{2}^{gt}\right)}^{2}$$

(7) $$A^{gt}=\left(x_{2}^{gt}-x_{1}^{gt}\right)*(y_{2}^{gt}-y_{1}^{gt})$$

(8) $$A^{prd}=\left(x_{2}^{prd}-x_{1}^{prd}\right)*(y_{2}^{prd}-y_{1}^{prd})$$

(9) $$x_{1}^{I}=\max\left(x_{1}^{prd},x_{1}^{gt}\right),x_{2}^{I}=\min\left(x_{2}^{prd},x_{2}^{gt}\right)$$

(10) $$y_{1}^{I}=\max\left(y_{1}^{prd},y_{1}^{gt}\right),y_{2}^{I}=\min\left(y_{2}^{prd},y_{2}^{gt}\right)$$

(11) $$I=\left\{\begin{array}{l} \left(x_{2}^{I}-x_{1}^{I}\right)*\left(y_{2}^{I}-y_{1}^{I}\right),if\;x_{2}^{I}>x_{1}^{I},y_{2}^{I}>y_{1}^{I}\\ 0\;otherwise\; \end{array}\right.$$

(12) $$IoU=\frac{I}{u},where\;u=A^{gt}+A^{prd}-I$$

(13) $$MPDIoU=IoU-\frac{d_{1}^{\;2}}{h^{2}+w^{2}}-\frac{d_{2}^{\;2}}{h^{2}+w^{2}}$$

(14) $$L_{MPDIoU}=1-MPDIoU$$

where:

- $\left(x_{1}^{prd},y_{1}^{prd},x_{2}^{prd},y_{2}^{prd}\right)$ are the coordinates of the predicted bounding box.
- $\left(x_{1}^{gt},y_{1}^{gt},x_{2}^{gt},y_{2}^{gt}\right)$ are the coordinates of the ground-truth bounding box.
- w is the width of the input image. h is the height of the input image.

## 3. Results and Discussion

This section outlines the experimental setup and specific details and subsequently provides an in-depth analysis and discussion of the experimental results.

### 3.1. Experimental Data

This study employed a dual-scheme approach for dataset construction, establishing a multi-source dataset by integrating data acquired online with field-collected samples. Recognizing that models trained on simple backgrounds often exhibit performance degradation in complex environments, particular emphasis was placed on augmenting the collection of disease samples under such challenging conditions.

Online data acquisition utilized a multi-source integration strategy. An automated web crawler system, guided by semantic labels such as “tea disease characteristics” and “tea disease spots,” performed targeted collection from platforms including Google Images and the CABI (Centre for Agriculture and Biosciences International) database. Simultaneously, plant disease data resources from platforms like Kaggle and Roboflow were incorporated. The initial dataset underwent a two-stage refinement process: first, blurry and low-resolution images were discarded; subsequently, manual screening was conducted to ensure the accuracy of disease labeling.

**Figure 6:**
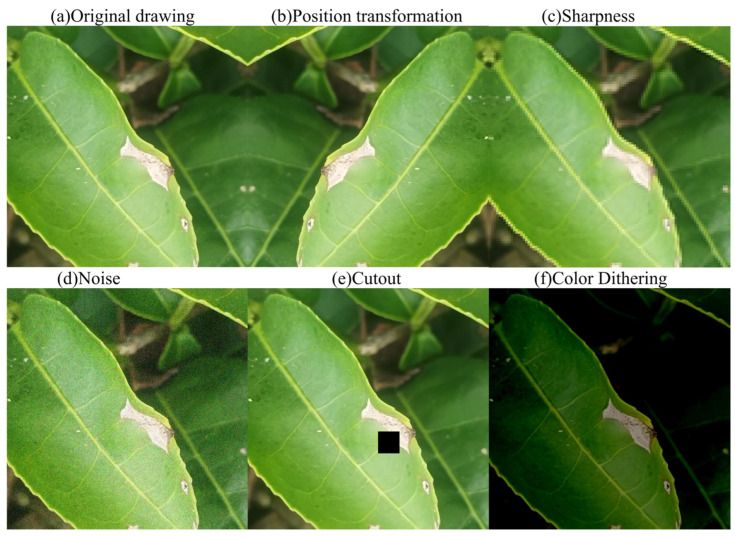
Data-enhanced image.

Field collection was undertaken in July and August 2024 at a tea garden in Huimin Town, Lancang Lahu Autonomous County, Pu’er City, Yunnan Province. High-resolution mobile devices were used for stereoscopic image acquisition across various time periods and under diverse lighting conditions to obtain original images of typical tea diseases. This field-collected data were then augmented using strategies such as random affine transformations and color space perturbations, resulting in an enhanced subset of 1500 field samples. This augmentation effectively improved the diversity of the data distribution and the model’s scene adaptability. The specific data augmentation methods are detailed in Table 1, and an example of an augmented image is presented in Figure 6.

**Figure 7:**
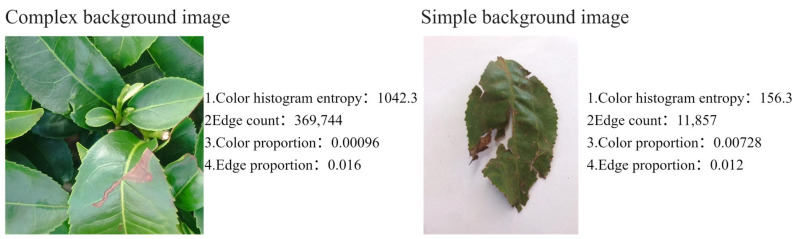
Example image for determining background complexity.

In this study, a ‘complex background’ is defined as a natural environment characterized by multiple visual features, including color heterogeneity, texture diversity, and morphological randomness. To establish a quantitative evaluation system for background complexity, an algorithm incorporating color region segmentation and edge detection was developed. This algorithm assesses image background complexity by quantifying the number of pixels within each distinct color region and along detected edges, subsequently applying an appropriate threshold for classification. An illustrative example of this process is provided in Figure 7.

The background complexity evaluation algorithm utilizes the following metrics:

- Color Histogram Entropy: This metric indicates the uniformity of color distribution. A low entropy value signifies a more concentrated color distribution, whereas a high entropy value suggests a more uniform distribution.
- Color Proportion: This refers to the ratio of pixels belonging to the most prevalent color in the entire image. A critical threshold of 60% is established; images exceeding this threshold are classified as having a simple background.
- Edge Proportion: This is the ratio of edge pixels to the total pixels in the image. Based on experimental results, a critical threshold of 1.5% is set. Images with an edge pixel proportion below this threshold are considered to have a simple background.

**Figure 8:**
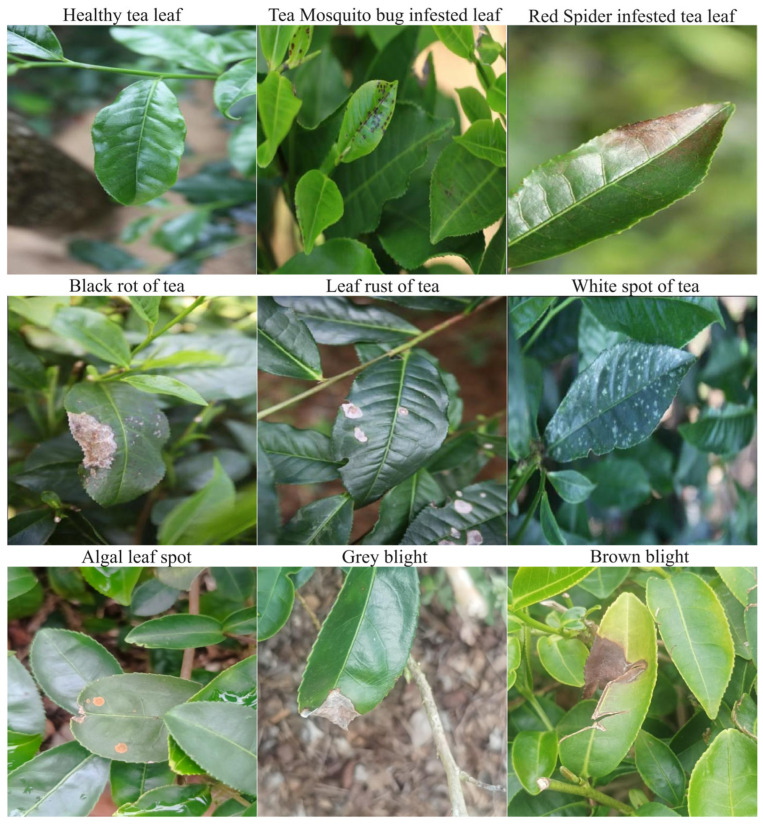
Self-made dataset Tea Datasets example diagram.

By integrating the multi-source data acquired online and through field collection and subsequently filtering it using the aforementioned background complexity evaluation algorithm, a standardized dataset, termed ‘Tea Datasets,’ was constructed. This dataset is designed to accurately reflect the characteristics of natural scenes. It encompasses eight typical tea diseases—namely; mosquito bites; red spider damage; black rot; leaf rust; white spot; algae leaf spot; gray blight; and brown blight—along with a healthy leaf control group. The total sample size is 6560 images, compiled over a data collection and processing period of two months. Sample images from this dataset are presented in Figure 8, and the specific distribution of samples across categories is detailed in Table 2.

In parallel, this study also utilized the public dataset ‘Tea Leaves Disease Datasets’ [34] to further validate the proposed model’s effectiveness and generalization capabilities. This dataset encompasses three common tea diseases: algal leaf spot, gray blight, and brown blight. The distribution of labels for each category within this public dataset is detailed in Table 3. By incorporating this public dataset and facilitating comparisons with existing methods, the performance of our model under diverse data conditions was evaluated, thereby providing a more comprehensive assessment of its robustness and applicability.

Tea disease detection in natural environments is significantly challenged by complex factors such as leaf occlusion, uneven illumination, and small lesion areas. Most existing public datasets are predominantly based on laboratory settings or simple backgrounds, failing to fully capture the diversity and complexity of disease presentation in real-world scenarios. For instance, dense leaf occlusion can obscure critical lesion features, while variations in light intensity may mask the texture information of low-contrast lesions. Furthermore, the accurate detection of minute lesions is crucial for effective early disease prevention and control.

**Figure 9:**
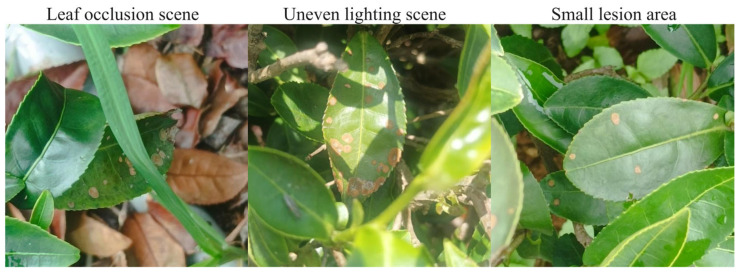
Example image of complex scene dataset.

To systematically evaluate the model’s robustness in practical applications, this study curated specific subsets from the self-constructed ‘Tea Datasets.’ An ‘occlusion subset’ was formed by selecting samples exhibiting leaf occlusion. An ‘illumination disturbance subset’ was created by choosing samples with uneven illumination and further augmented by programmatically adjusting image brightness and contrast. Finally, a ‘small lesion subset’ was compiled by selecting images based on the proportional area of the lesions. These subsets were then combined to construct a comprehensive ‘complex scene dataset’ that encompasses instances of occlusion, uneven illumination, and small lesions. The specific distribution of samples within this dataset is detailed in Table 4, and representative images are presented in Figure 9.

### 3.2. Experimental Details

The experimental platform for this study utilized the Ubuntu 22.04 LTS operating system. Hardware specifications included an AMD Ryzen 5 5600X processor, 16 GB of DDR4 RAM, and an AMD Radeon RX 6700 XT graphics card with 10 GB of VRAM. The PyTorch 2.5.0 deep learning framework was employed. All input images from both the self-built and public datasets were resized to a fixed resolution of 640 × 640 pixels before being fed into the models for both training and evaluation. Training configuration parameters were set as follows: 150 epochs, a batch size of 32, an initial learning rate of 1 × 10^−2^, a momentum coefficient of 0.937, and a weight decay coefficient of 5 × 10^−4^. The Stochastic Gradient Descent (SGD) optimizer was used. The dataset was partitioned into training (70%), validation (20%), and test (10%) sets, following a 7:2:1 ratio.

Model evaluation was performed using a comprehensive suite of quantitative metrics, including

Precision: The proportion of correctly identified positive samples among all samples predicted as positive. It is calculated as

(15) $$Precison=\frac{TP}{TP+FP}\times 100\%$$

Recall: The proportion of correctly identified positive samples among all actual positive samples. It is calculated as

(16) $$Recall=\frac{TP}{TP+FN}\times 100\%$$

Mean Average Precision (mAP): The arithmetic mean of Average Precision (AP) values across all classes. It is calculated as

(17) $$mAP=\frac{\sum_{i=1}^{c}AP(c)}{C}\times 100\%$$

where C is the total number of classes (9 and 3, respectively, for the datasets in this study).

Complexity: Measured in Giga Floating-point Operations Per Second (GFLOPs) to quantify the model’s computational load.

Model Size: Assessed by the total number of parameters and the storage space occupied (e.g., in megabytes, MB).

### 3.3. Ablation Experiment

**Figure 10:**
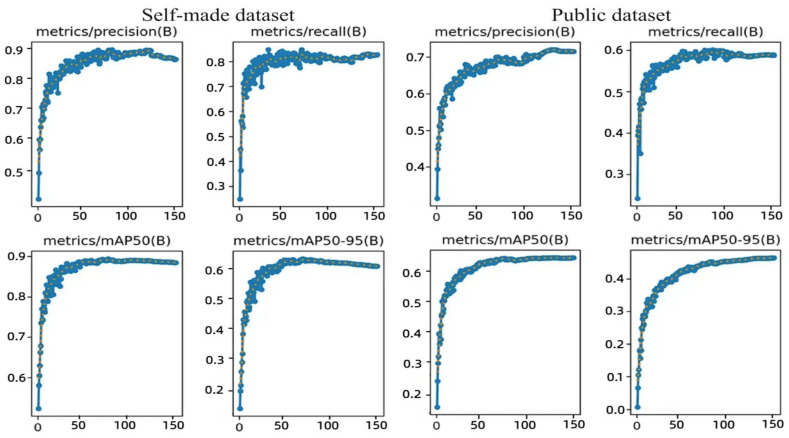
Performance metrics curve of YOLO-SSM.

This study implemented structural optimizations within the YOLOv8n architecture to enhance detection performance. To validate the efficacy of these modifications, ablation experiments were conducted, and the results are presented in Table 5 and Table 6. Figure 10 illustrates the progression of the accuracy curve throughout the training process. The training performance metrics stabilized after approximately 150 iterations, indicating that the model successfully achieved convergence.

Both our proposed YOLO-SSM and the baseline YOLOv8n were trained for 150 epochs, with performance analyzed via precision, recall, mAP@0.5, and computational complexity.

Ablation studies on our self-built dataset (Table 5) revealed specific module contributions. Replacing CIoU with MPDIoU yielded a 2.5% mAP increase, significantly enhancing bounding box prediction accuracy and stability, especially for localizing small, indistinct early-stage tea lesions. SSPDConv alone contributed a 2.2% mAP rise by bolstering feature expression and suppressing common field-imagery background interference through its SPD transformation and SimAM attention, effectively capturing finer lesion details despite a slight increase in computational load. ESPPFCSPC added a 2.3% mAP improvement, demonstrating its effectiveness in expanding the receptive field with dilated convolution to capture broader spatial context, beneficial for varied lesion sizes and partially obscured targets in dense tea canopies. Cumulatively, the integrated YOLO-SSM achieved an 89.8% mAP on this dataset, a significant 3.9% total improvement over YOLOv8n, justifying the 1.1 GFLOPs computational increase with its enhanced ability to detect challenging tea diseases through combined strengths in detail preservation (SSPDConv), multi-scale understanding (ESPPFCSPC), and precise small target localization (MPDIoU).

On the public dataset (Table 6), SSPDConv again demonstrated a significant impact, improving mAP by 3.2% due to its enhanced adaptability to diverse complex backgrounds via dynamic feature fusion, with a moderate 1.0 GFLOPs increase. ESPPFCSPC contributed a 2.4% mAP gain by optimizing multi-scale feature aggregation. MPDIoU yielded a 0.9% mAP improvement; while modest in aggregate here, its primary strength in enhancing localization robustness for minute objects remains crucial for early disease detection. The final YOLO-SSM achieved 68.5% mAP on this public dataset, a notable 4.3% improvement over YOLOv8n. This gain, with a manageable 1.1 GFLOPs increase, underscores the synergistic benefits of the modules in handling complex backgrounds, multi-scale features, and localization, justifying the moderate complexity increase for the achieved accuracy vital for disease identification.

Further comparative validation of the improved modules against their original counterparts confirmed their benefits. SSPDConv improved mAP by 0.6% and 0.7% over SPDConv, with enhanced recall, particularly for small/occluded targets, attributable to SimAM, without significant computational rise on the public set. ESPPFCSPC, using dilated convolution, improved mAP by 0.5% and 0.9% over SPPFCSPC, showing better adaptability to complex backgrounds with stable computational load on the self-built set. In essence, SSPDConv boosts recall for target-dense scenes, while ESPPFCSPC effectively balances accuracy and efficiency.

Overall, YOLO-SSM demonstrated significant accuracy improvements on both datasets. MPDIoU showed more prominent optimization effects on the self-built dataset, while SSPDConv’s dynamic feature fusion was more adaptable on the public dataset. Despite a slight increase in computational load from module stacking, YOLO-SSMs balanced design surpasses YOLOv8n in overall performance, validating the modules’ generalization and effectiveness.

In summary, the meticulously designed YOLO-SSM architecture enhanced detection accuracy, achieving 3.9% and 4.3% mAP improvements over the baseline on self-built and public datasets, respectively, with its efficacy validated by systematic ablation experiments.

### 3.4. Comparative Experiment

To further ascertain the superiority of the YOLO-SSM model, this study conducted comparative experiments against contemporary state-of-the-art models. Specifically, the advanced tea disease detection model YOLO-RMDA, previously cited, and the high-performance model PP-YOLOE [35], which is well-suited for complex backgrounds, were reproduced for this comparison. PP-YOLOE is a high-performance, single-stage object detection model that enhances feature extraction capabilities through deformable convolution and introduces an adaptive feature fusion module to dynamically adjust multi-scale feature weights. These features significantly improve its detection performance for small and occluded targets, demonstrating excellent efficacy in complex background scenarios. The performance of YOLO-SSM was benchmarked against YOLO-RMDA, PP-YOLOE, YOLOv11n [36], and other classic mainstream object detection models on both datasets. The comparative results are presented in Table 7 and Table 8.

As evidenced in Table 7 and Table 8, YOLO-SSM demonstrates significant comprehensive advantages when compared to contemporary high-performance models.

On the self-made dataset, YOLO-SSM achieved an mAP of 89.7%, surpassing YOLO-RMDA by 3.6%, with corresponding increases in precision and recall of 7.7% and 6.9%, respectively. On the public dataset, its mAP was 0.7% higher than that of YOLO-RMDA, and precision increased by a notable 10.8%, validating the strong generalization capability of YOLO-SSM for complex disease characteristics. However, the computational complexity of YOLO-SSM is 4.3 GFLOPs higher than YOLO-RMDA, and its parameter count is also larger, indicating that YOLO-RMDA maintains an advantage in extreme lightweight scenarios.

When compared with PP-YOLOE, YOLO-SSM exhibited mAP improvements of 1.5% and 0.3% on the self-made and public datasets, respectively, while concurrently reducing both the number of parameters and computational load. This highlights the efficiency of YOLO-SSMs lightweight design. Although PP-YOLOE achieves a higher recall rate by leveraging a powerful backbone network, its substantial computational complexity severely limits its potential for edge deployment. In contrast, YOLO-SSM strikes a balance between accuracy and efficiency through the detail preservation mechanism of SSPDConv and the multi-scale feature extraction capabilities of ESPPFCSPC.

YOLO-SSM also performs commendably against other high-performance models proposed in recent years. Compared to YOLOv11n, its mAP improved by 3% on both the self-made and public datasets, with an associated increase in precision, suggesting that the MPDIoU loss function offers more accurate localization of small targets. Relative to the baseline YOLOv8n, YOLO-SSM improved mAP by 3.8% and 2.7% on the self-made and public datasets, respectively, with only a marginal increase in computational complexity, thereby proving the efficiency of its dynamic feature fusion strategy.

In comparison with classic models, YOLO-SSM effectively overcomes the traditional accuracy limitations of lightweight architectures. For instance, its mAP on the self-made dataset increased by 4.6% compared to YOLOv5s. Against SSD, YOLO-SSM achieved a substantial mAP improvement of 18.6% on the self-made dataset while also reducing model size. Furthermore, compared to Faster R-CNN, YOLO-SSM increased mAP by 16.5% on the self-made dataset with a significantly smaller computational workload, fully reflecting the accuracy and efficiency advantages of single-stage models in agricultural applications.

While the proposed YOLO-SSM model introduces additional modules leading to a moderate increase in computational complexity, the achieved mAP improvements of 3.9% and 4.3% on the self-made and public datasets, respectively, represent a worthwhile trade-off for the challenging domain of in-field tea disease detection. The significance of these gains is further underscored by the model’s enhanced ability to detect small and occluded targets, which are critical for early disease intervention and often missed by less complex models.

**Figure 11:**
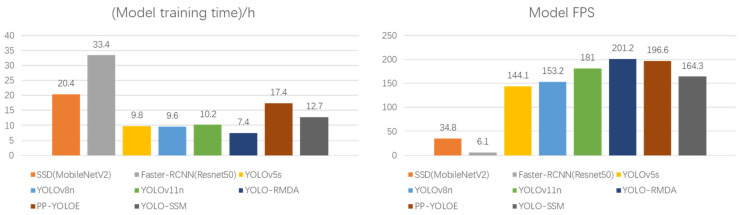
Model training time and FPS.

Further analysis, in conjunction with Figure 11, reveals that YOLO-SSM achieves higher accuracy with a comparatively low computational workload. This is attributed to the synergistic combination of the SSPDConv module, which incorporates an attention mechanism, and the dual-channel ESPPFCSPC spatial pyramid pooling structure. The model’s inference speed and training time are maintained within a deployment-friendly range, verifying its potential for deployment on edge devices. The experimental results collectively demonstrate that YOLO-SSM has reached an advanced level in terms of accuracy, robustness, and engineering applicability, offering an efficient and reliable solution for agricultural disease detection.

### 3.5. Model Robustness Analysis and Performance Verification in Complex Scenes

To assess the practical detection capabilities of YOLO-SSM in complex natural environments, this study utilized a self-constructed dataset specifically designed to represent such challenging scenarios. This ‘complex scene dataset’ encompasses key interference factors, including leaf occlusion, uneven illumination, and small lesion areas. Through experiments on this dataset, a systematic analysis was conducted to identify the core factors that significantly impact model performance in real-world application scenarios. The experimental results are presented in Table 9.

In occlusion scenarios, where tea leaves frequently overlap and obscure crucial lesion details, YOLO-SSM achieved an mAP of 82.1%, surpassing the baseline YOLOv8n model by 4.2%. Both precision and recall rates were simultaneously optimized, indicating that the SSPDConv module effectively mitigates detail loss caused by leaf occlusion. This is attributed to its space-to-depth conversion and SimAM attention mechanism, which validate its capability to retain local features. In scenes with uneven illumination, a common challenge arising from sunlight reflection on glossy tea leaves and shadows cast by dense foliage, the mAP of YOLO-SSM increased to 74.5%, a significant improvement of 6.8% over YOLOv8n. The ESPPFCSPC module, by employing dilated convolution, expands the receptive field and adaptively enhances the contextual feature expression of low-contrast lesions, thereby significantly improving detection stability. For small lesion areas, which represent critical early stages of tea diseases often indistinguishable or missed by general detectors in field conditions, YOLO-SSM achieved a recall rate of 67.2%, a substantial increase of 16.1% compared to YOLOv8n. This validates that the MPDIoU loss function, through its multi-point distance optimization strategy, effectively enhances sensitivity for small target localization and addresses the issue of missed detections for minute lesions. These experimental results collectively demonstrate that YOLO-SSM exhibits superior adaptability and reliability in actual complex scenes, providing robust technical support for the accurate detection of tea diseases.

### 3.6. Cross-Validation Experiment

To further evaluate the stability and generalization performance of the proposed YOLO-SSM model, a 5-fold cross-validation experiment was conducted on the self-built ‘Tea Datasets’. In this procedure, the dataset was randomly partitioned into five equally sized folds. For each of the five iterations, one distinct fold was reserved as the test set, while the remaining four folds were utilized for training the model. Consistent hyperparameters, as detailed in Section 3.2, were maintained across all folds, and no fold-specific hyperparameter tuning was performed, ensuring an unbiased assessment of the model’s inherent robustness.

The comprehensive results of this 5-fold cross-validation are presented in Table 10. The YOLO-SSM model demonstrated a high degree of consistency in its performance across the different data partitions. Specifically, the precision values ranged from 90.3% to 90.8%, mAP@0.5 values from 89.4% to 89.8%, and recall rates from 83.9% to 84.6%.

The average performance metrics across these five folds were an average precision of 90.6% (±0.19%), an average mAP@0.5 of 89.6% (±0.16%), and an average recall of 84.2% (±0.28%). The minimal variation in performance across the folds, as indicated by the low standard deviations, underscores the stability and robust generalization capabilities of the YOLO-SSM model. This cross-validation approach effectively mitigates potential biases that might arise from a single, fixed data split and provides a more comprehensive and reliable assessment of the model’s practical effectiveness in the context of tea disease detection.

### 3.7. Visual Comparison Verification

**Figure 12:**
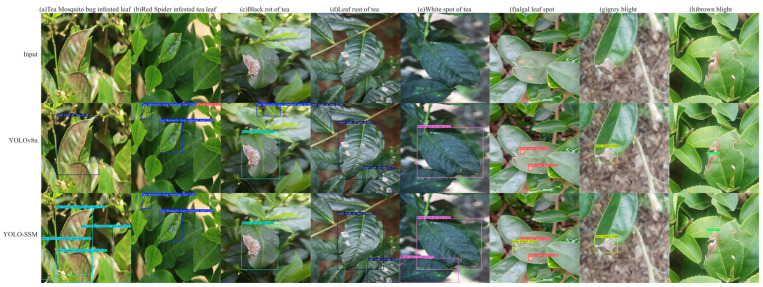
Comparison of actual detection results of different algorithms.

Visual analysis offers a spatial mapping basis for verifying algorithm performance. By visualizing the spatial coordinates, size parameters, and classification labels of the target bounding boxes, the spatial resolution capabilities and decision reliability of the detection system can be intuitively assessed [37]. As illustrated in Figure 12, several observations can be made:

As illustrated in Figure 12, a visual comparison of detection results reveals several key performance differences between YOLO-SSM and the baseline YOLOv8n model:

- Figure 12a: In a scenario with mosquito-bitten tea leaves, YOLO-SSM successfully detected all instances. In contrast, the baseline model misclassified these mosquito-bitten leaves as leaf rust.
- Figure 12b–d: For these samples, the improved YOLO-SSM model accurately identified all true diseased areas, whereas the baseline model produced false detection by incorrectly identifying healthy leaf portions or non-disease artifacts as diseased.
- Figure 12e: The baseline model failed to detect a leaf exhibiting white spot disease, which YOLO-SSM correctly identified.
- Figure 12f: In a complex scenario involving multiple co-occurring disease types, the baseline YOLOv8n model missed certain disease spots, detecting only the algae leaf spot area. While YOLO-SSM successfully detected the gray blight area, it did miss a small target area of algae leaf spot. This particular missed detection for algae leaf spot by YOLO-SSM is likely attributable to an insufficient number of training samples for this specific disease, leading to suboptimal feature extraction.
- Figure 12g: For the gray blight detection image, YOLO-SSM demonstrated a higher prediction confidence score compared to the baseline model for the correctly identified disease.
- Figure 12h: YOLO-SSM was able to completely detect the diseased area of brown blight, while the baseline model only identified a portion of it.

The visual comparisons highlight the superior robustness of our proposed YOLO-SSM algorithm, particularly under challenging conditions with complex backgrounds. This enhanced performance, evident in its ability to accurately identify subtle, overlapping, or partially occluded diseased areas, is driven by three key innovations: The SSPDConv module enhances feature discrimination, the ESPPFCSPC module improves feature map resolution and information integrity, and the MDPIoU loss function refines the localization of small targets.

**Figure 13:**
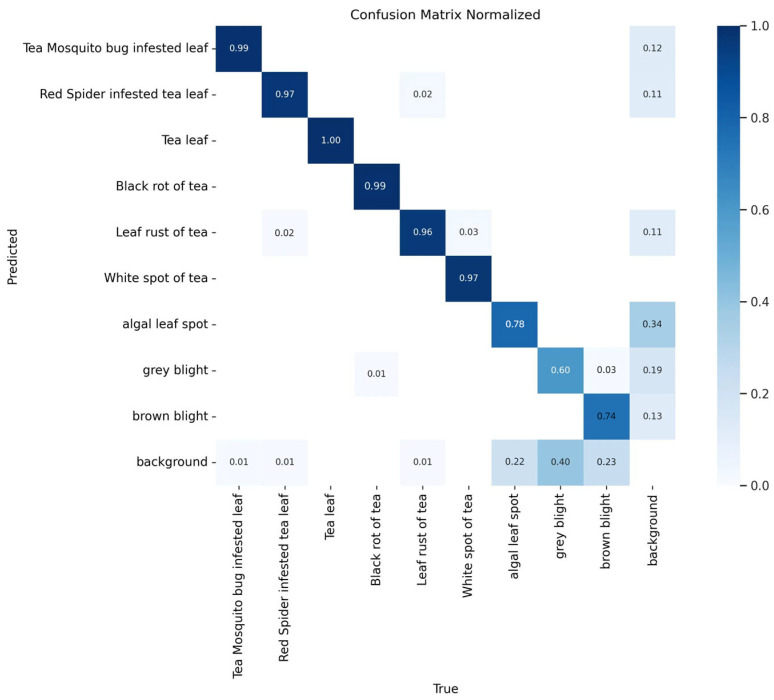
Confusion Matrix Normalized.

To assess the YOLO-SSM model’s proficiency in identifying individual tea diseases, a confusion matrix was employed for visual evaluation and analysis. In a confusion matrix, diagonal elements represent instances where the model correctly detected and classified the disease. Conversely, off-diagonal elements indicate targets that were either not detected (missed detections) or misclassified. The intensity of color along the diagonal corresponds to the prediction accuracy for each respective category. The confusion matrix for the YOLO-SSM model evaluated on the self-made dataset is presented in Figure 13.

As depicted in Figure 13, the YOLO-SSM model demonstrated excellent detection performance for three specific diseases: Tea leaves bitten by mosquitoes, tea leaves infested by red spiders, and leaf rust. This high accuracy is likely attributable to the relatively large lesion areas and distinct disease characteristics associated with these conditions, resulting in very few missed detections or false positives by the model. The model also exhibited excellent detection efficacy for black rot and white spot disease. This particular observation might be influenced by the smaller number of samples for black rot and white spot disease in the dataset, potentially leading to a degree of overfitting for these categories. Future studies could further validate the model’s identification capabilities for these two diseases by augmenting the dataset with more samples.

Conversely, the model showed comparatively poorer detection results for algal leaf spot, gray blight, and brown blight, with instances of both false positives and missed detections. This reduced performance is likely due to the smaller sample sizes for these three diseases in the self-made dataset, coupled with the fact that their lesion spots are often small and share similar visual characteristics. Consequently, this study introduced a public dataset to further evaluate the model’s ability to identify these particular diseases. Although the YOLO-SSM model did not achieve perfect detection across all categories, it accurately identified the majority of tea diseases, underscoring its overall effectiveness in the context of tea disease detection.

**Figure 14:**
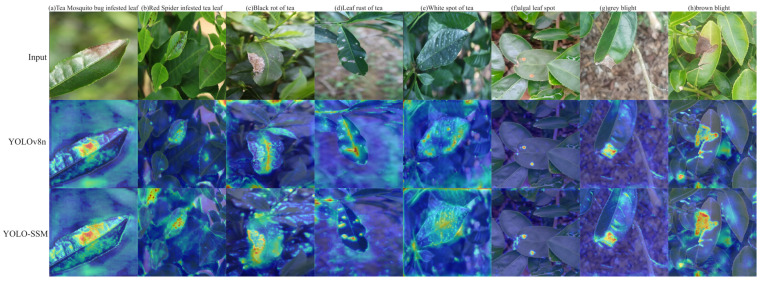
Grad-CAM visualization results of different algorithms.

To investigate the interpretability of the model’s decision-making mechanism, this study employed the Grad-CAM (Gradient-weighted Class Activation Mapping) technique [38] to visualize network feature responses. The resulting heatmaps are presented in Figure 14. A comparative analysis of these heatmaps reveals that YOLO-SSM exhibits significantly more concentrated attention in regions corresponding to disease locations. The activation intensity within these key areas is higher for YOLO-SSM compared to the baseline model, and there is a greater spatial overlap with the actual pathological feature areas. This enhanced focus is attributed to the attention guidance provided by the SSPDConv module and the feature refinement capabilities of the ESPPFCSPC module, thereby validating the effectiveness of these improved modules in enhancing model interpretability and suppressing background noise.

Observing Figure 14a–c, the heatmaps generated by the baseline model fail to completely encompass the diseased areas. In contrast, the heatmaps from YOLO-SSM accurately focus on these regions. This indicates that the YOLO-SSM model, through its SSPDConv convolutional structure combined with the attention mechanism, effectively reduces interference from complex backgrounds, enabling the model to concentrate more precisely on the diseased areas.

Further analysis of Figure 14d–f reveals that while both models can generally locate the diseased areas, the baseline model allocates less attention to the critical regions and exhibits instances of false background activation. Conversely, YOLO-SSM demonstrates significantly greater attention to the key diseased areas and a lower false activation rate for non-diseased background regions. These experiments collectively show that YOLO-SSM effectively suppresses complex background interference and achieves precise focus on diseased areas, thereby verifying its advantages in feature selection and noise immunity.

### 3.8. Error Analysis

To further probe the differences in model behavior, a qualitative error analysis was performed, focusing on instances where the baseline YOLOv8n model failed and YOLO-SSM succeeded, or vice versa.

**Figure 15:**
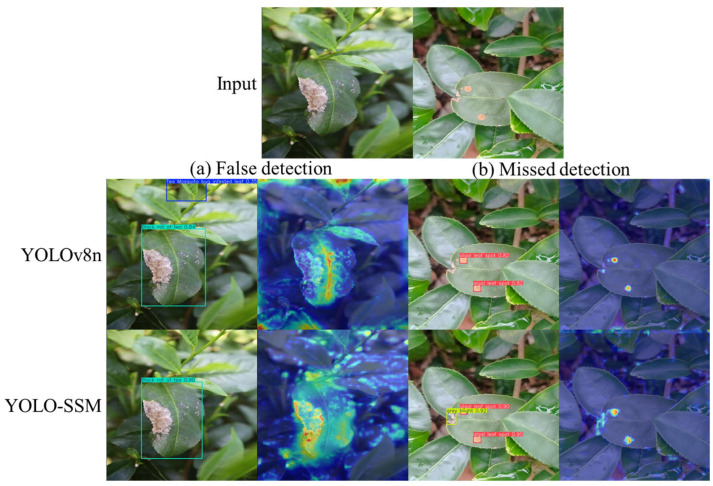
Comparison of YOLOv8n and YOLO-SSM on detections.

Figure 15, a comparison of YOLOv8n and YOLO-SSM on detections, illustrates typical error cases. Figure 15a presents a scenario where YOLOv8n incorrectly identified a leaf as a Tea Mosquito bug-infested leaf (false detection). The Grad-CAM for YOLOv8n indicates strong activation on this non-disease element. YOLO-SSM, however, avoided this false detection, and its Grad-CAM shows minimal activation in that area, indicating better discrimination against background noise and confusing visual patterns. In Figure 15b, YOLOv8n failed to detect a small grey blight lesion. The corresponding Grad-CAM visualization for YOLOv8n shows weak and unfocused activation in the target region. In contrast, YOLO-SSM correctly identified this lesion, and its Grad-CAM clearly highlights concentrated attention on the lesion, demonstrating its superior sensitivity to such subtle features. These targeted visualizations underscore how the architectural modifications in YOLO-SSM lead to more reliable feature learning and attention allocation, particularly in challenging cases.

To provide a more granular and insightful analysis of the performance gains offered by YOLO-SSM, we conducted a detailed error diagnosis inspired by the TIDE (A Toolkit for Identifying Detection Error) [39] framework. This approach decomposes the overall error into specific components, allowing us to understand not just that our model is better, but why and how it improves upon the baseline. The analysis focuses on key error types, including classification error ($E_{cls}$), localization error ($E_{loc}$), background error ($E_{bkg}$), missed detection error ($E_{miss}$), false positives ($E_{FP}$), and false negatives ($E_{FN}$). The comparative results on the self-built dataset are presented in Table 11.

As detailed in Table 11, a TIDE-inspired error analysis confirms that YOLO-SSMs +3.8 mAP50 improvement stems from systematic error reduction. The most substantial gain is a +7.7 point decrease in False-Positive errors, indicating superior precision. This enhanced ability to suppress background interference is primarily attributed to the SSPDConv module’s integrated SimAM attention.

Crucially, YOLO-SSM also reduces Missed Detection errors by +2.8 points, a vital improvement for a reliable diagnostic tool. This enhanced sensitivity is driven by the MPDIoU loss function, which aids small-target localization, and SSPDConv’s detail preservation. Furthermore, the +1.3 points reduction in Localization error further validates MPDIoUs effectiveness in producing tighter bounding boxes. Modest but important reductions in Classification and Background errors suggest the synergistic combination of SSPDConv and ESPPFCSPC yields richer, more discriminative features.

In conclusion, this analysis demonstrates that YOLO-SSMs accuracy gains are achieved through targeted architectural enhancements that systematically reduce false alarms, missed detections, and localization errors for the challenging task of tea disease identification.

## 4. Conclusions

This paper introduced YOLO-SSM, a model developed through targeted improvements and optimizations of the YOLOv8n architecture. Specifically, the integration of the SSPDConv convolution module enhanced the model’s information extraction capabilities and allowed for the capture of richer spatial context information. The original model’s spatial pyramid pooling structure was optimized by employing the dual-channel ESPPFCSPC module for feature extraction, thereby refining multi-scale feature expression and further augmenting feature representation capabilities. Additionally, the loss function was replaced with MPDIoU to improve the model’s target localization accuracy, leading to more precise optimization of prediction results.

Through comprehensive training on both self-constructed and public datasets, complemented by extensive experimental validation, visualization analysis, and thorough comparisons with mainstream and baseline models, the proposed YOLO-SSM model demonstrated significant enhancements in object detection performance. Notably, YOLO-SSM achieved a mean Average Precision (mAP@0.5) of 89.8% on our self-built ‘Tea Datasets’ and 68.5% on the public ‘Tea Leaves Disease Datasets,’ representing substantial improvements of 3.9% and 4.3%, respectively, over the baseline YOLOv8n model. These quantitative gains underscore its superior capability, particularly in addressing the specific challenges of leaf occlusion, uneven illumination, and small target detection prevalent in complex tea plantation backgrounds. This includes improved detection of occluded targets and small targets, thereby confirming the overall effectiveness of the model for practical tea disease management and agricultural applications. Furthermore, while the primary focus of this work was to optimize a model for tea disease detection, future research could explore the generalizability of the proposed SSPDConv and ESPPFCSPC modules on broader object detection benchmarks, such as COCO, to assess their potential for wider applicability.

Looking ahead, integrating multi-modal data presents a promising frontier for advancing tea disease detection. Advanced fusion techniques that combine visual information with other data modalities can significantly enhance system robustness and accuracy. Inspiration can be drawn from sophisticated architectures like ‘Divide-and-Conquer’ triple-flow networks [40] used in RGB-Thermal salient object detection. Such strategies effectively manage inter-modality discrepancies and improve resilience to noisy sensor inputs by concurrently processing modality-specific and complementary information streams. Adapting these principles—potentially through specialized feature modulators or dynamic aggregation modules for tea leaf imagery combined with other sensor data—could yield more comprehensive and robust diagnostic systems for tea diseases; especially in challenging field conditions.

## Figures and Tables

**Table 1:** Data augmentation methods.

Enhancement	Operation
Position transformation	Random rotation, Image translation, Mirror flipping
Sharpness	Adjusts image clarity
Noise	Generate noise
Cutout	Randomly occludes a portion of the image
Color Dithering	Random chromaticity, Saturation, and brightness enhancement

**Table 2:** The number of labels for each disease in the self-made Tea Dataset.

Index	Classes	Number of Images
1.	healthy tea leaf	276
2	Tea Mosquito bug infested leaf	3193
3	Red Spider infested tea leaf	395
4	Black rot of tea	61
5	Leaf rust of tea	1289
6	White spot of tea	193
7	algal leaf spot	481
8	grey blight	406
9	brown blight	471

**Table 3:** The number of labels for each disease in the publicly available tea leaves disease dataset.

Index	Classes	Number of Images
1	algal leaf spot	1392
2	grey blight	981
3	brown blight	1074

**Table 4:** The number of labels for each disease in the public Tea leaves disease dataset.

Index	Classes	Number of Images
1	Leaf shading	200
2	Uneven light	200
3	Small diseased area	100

**Table 5:** Ablation experiments on self-made datasets.

Model	Precision/%	mAP50/%	Recall/%	Parameters/M	GFLOPs	Model Size/MB
YOLOv8n	0.868	0.859	0.798	3.0	8.1	6.3
YOLOv8n + SPDConv	0.874	0.875	0.839	3.6	9.0	6.5
YOLOv8n + SSPDConv	0.879	0.881	0.845	3.7	9.1	6.6
YOLOv8n + SPPFCSPC	0.882	0.877	0.831	3.9	8.9	6.4
YOLOv8n + ESPPFCSPC	0.891	0.882	0.833	3.9	8.9	6.4
YOLOv8n + MPDIou	0.878	0.884	0.845	3.0	8.1	6.4
YOLO-SSM	0.908	0.898	0.842	4.0	9.2	6.8

**Table 6:** Ablation experiments on public datasets.

Model	Precision/%	mAP50/%	Recall/%	Parameters/M	GFLOPs	Model Size/MB
YOLOv8n	0.689	0.642	0.571	3.0	8.1	6.3
YOLOv8n + SPDConv	0.653	0.667	0.665	3.6	9.0	6.5
YOLOv8n + SSPDConv	0.657	0.674	0.678	3.7	9.1	6.6
YOLOv8n + SPPFCSPC	0.691	0.657	0.613	3.9	8.9	6.4
YOLOv8n + ESPPFCSPC	0.698	0.666	0.627	3.9	8.9	6.4
YOLOv8n + MPDIou	0.689	0.651	0.560	3.0	8.1	6.4
YOLO-SSM	0.743	0.685	0.601	4.0	9.2	6.8

**Table 7:** Comparison and experimental verification results of different algorithms on self-made datasets.

Model	Precision/%	mAP50/%	Recall/%	Parameters/M	GFLOPs	Model Size/MB
Faster-RCNN(Resnet50)	85.8	73.2	53.2	25.5	215.6	102
SSD(MobileNetV2)	85.3	71.1	55.2	3.4	3.3	13.6
YOLOv5s	81.6	85.1	79.3	2.5	7.1	5.3
YOLOv8n	82.3	85.9	78.1	3.0	8.1	6.3
YOLOv11n	86.9	86.7	79.9	2.6	6.3	5.5
YOLO-RMDA	83.1	86.1	77.3	1.8	4.9	4.6
PP-YOLOE	85.4	88.2	81.1	7.8	16.9	7.9
YOLO-SSM	90.8	89.7	84.2	4.0	9.2	6.8

**Table 8:** Comparison and experimental verification results of different algorithms on public datasets.

Model	Precision/%	mAP50/%	Recall/%	Parameters/M	GFLOPs	Model Size/MB
Faster-RCNN(Resnet50)	64.3	58.6	51.6	25.5	215.6	102
SSD(MobileNetV2)	56.8	52.8	50.2	3.4	3.3	13.6
YOLOv5s	60.3	63.5	61.6	2.5	7.1	5.3
YOLOv8n	61.9	65.8	60.9	3.0	8.1	6.3
YOLOv11n	70.1	65.5	57.6	2.6	6.3	5.5
YOLO-RMDA	63.5	67.8	61.2	1.8	4.9	4.6
PP-YOLOE	71.9	68.2	62.8	7.8	16.9	7.9
YOLO-SSM	74.3	68.5	60.1	4.0	9.2	6.8

**Table 9:** Experimental verification results of different algorithms on complex datasets.

Scene	Model	Precision/%	mAP50/%	Recall/%
Leaf shading	YOLOv8n	79.8	77.9	68.8
	YOLO-SSM	82.5	82.1	73.9
Uneven light	YOLOv8n	71.4	67.7	60.1
	YOLO-SSM	76.1	74.5	69.2
Small diseased area	YOLOv8n	65.5	62.1	51.1
	YOLO-SSM	72.4	70.3	67.2

**Table 10:** Cross-validation experimental results of self-made datasets.

Folds	Precision/%	mAP50/%	Recall/%
1	90.8	89.8	84.3
2	90.7	89.6	84.1
3	90.3	89.7	84.6
4	90.5	89.4	83.9
5	90.6	89.5	84.0
Average	90.6	89.6	84.2
Std Dev	0.19	0.16	0.28

**Table 11:** TIDE-Inspired Error Analysis: Comparison of YOLOv8n and YOLO-SSM on the Self-Made Dataset.

Model	mAP50↑	$E_{cls}↓$	$E_{loc}↓$	$E_{bkg}↓$	$E_{miss}↓$	$E_{FP}↓$	$E_{FN}↓$
YOLOv8n	85.9	4.1	5.5	3.8	18.6	16.9	18.6
YOLO-SSM	89.7	3.5	4.2	2.9	15.8	9.2	15.8
Improvement	+3.8	+0.6	+1.3	+0.9	+2.8	+7.7	+2.8

## Data Availability

The datasets used and analyzed during the current study are available from the corresponding author upon reasonable request.

## Abbreviations

The following abbreviations are used in this manuscript:

YOLO	You Only Look Once
YOLOv8	You Only Look Once version 8
SVM	Support Vector Machines
KNN	K-Nearest Neighbor algorithms
CNN	Convolutional Neural Network
SSD	Single Shot Multibox Detector
YOLO-SSM	YOLOv8 incorporating SSPDConv, ESPPFCSPC, and MPDIoU
ESPPFCSPC	Enhanced Spatial Pyramid Pooling Fast with Cross Stage Partial connection
C2f	Cross Stage Partial 2 with Focus
FPN	Feature Pyramid Network
PANet	Path Aggregation Network
SimAM	A Simple, Parameter-Free Attention Module
SPPF	Spatial Pyramid Pooling Fast
Conv	Convolution
SPD	space-to-depth
CSP	Cross Stage Partial
CIoU	Complete Intersection over Union
MPDIoU	Multi-Point Distance Intersection over Union
CABI	Centre for Agriculture and Biosciences International
SGD	Stochastic Gradient Descent
mAP	Mean Average Precision
GFLOPs	Giga Floating-point Operations Per Second
Grad-CAM	Gradient-weighted Class Activation Mapping
